# Replication study of 34 common SNPs associated with prostate cancer in the Romanian population

**DOI:** 10.1111/jcmm.12729

**Published:** 2016-01-15

**Authors:** Viorel Jinga, Irma Eva Csiki, Andrei Manolescu, Paul Iordache, Ioan Nicolae Mates, Daniel Radavoi, Stefan Rascu, Daniel Badescu, Paula Badea, Dana Mates

**Affiliations:** ^1^ “Prof. Dr. Th. Burghele” Clinical Hospital Urology Department University of Medicine and Pharmacy “Carol Davila” Bucharest Romania; ^2^ National Institute of Public Health Bucharest Romania; ^3^ School of Science and Engineering Reykjavik University Reykjavik Iceland; ^4^ “St Mary” Clinical Hospital General Surgery Department University of Medicine and Pharmacy “Carol Davila” Bucharest Romania

**Keywords:** prostate cancer, single nucleotide polymorphisms, replication

## Abstract

Prostate cancer is the third‐most common form of cancer in men in Romania. The Romanian unscreened population represents a good sample to study common genetic risk variants. However, a comprehensive analysis has not been conducted yet. Here, we report our replication efforts in a Romanian population of 979 cases and 1027 controls, for potential association of 34 literature‐reported single nucleotide polymorphisms (SNPs) with prostate cancer. We also examined whether any SNP was differentially associated with tumour grade or stage at diagnosis, with disease aggressiveness, and with the levels of PSA (prostate specific antigen). In the allelic analysis, we replicated the previously reported risk for 19 loci on 4q24, 6q25.3, 7p15.2, 8q24.21, 10q11.23, 10q26.13, 11p15.5, 11q13.2, 11q13.3. Statistically significant associations were replicated for other six SNPs only with a particular disease phenotype: low‐grade tumour and low PSA levels (rs1512268), high PSA levels (rs401681 and rs11649743), less aggressive cancers (rs1465618, rs721048, rs17021918). The strongest association of our tested SNP's with PSA in controls was for rs2735839, with 29% increase for each copy of the major allele G, consistent with previous results. Our results suggest that rs4962416, previously associated only with prostate cancer, is also associated with PSA levels, with 12% increase for each copy of the minor allele C. The study enabled the replication of the effect for the majority of previously reported genetic variants in a set of clinically relevant prostate cancers. This is the first replication study on these loci, known to associate with prostate cancer, in a Romanian population.

## Introduction

In Romania, prostate cancer is the third‐most common form of cancer in males (after lung and colon cancers) with an age‐standardized incidence rate per 100,000 estimated to 37.9 in 2012. This incidence rate is among the lowest in Europe and reflects the absence of a routinely PSA screening programme for prostate cancer. Instead, PSA testing is recommended to the men having suggestive signs and symptoms of prostate cancer and is followed by additional examinations (DRE‐digital rectal exam, ultrasound, biopsy). The vast majority of incident cases have advanced disease at diagnosis carrying a poor prognosis and inevitably resulting in death; the age‐standardized mortality rate is estimated to 16.3 per 100,000 in 2012 1.

Prostate cancer does not have any strong environmental risk factors but there is a great body of evidence that genetic factors may contribute more than 60% of the population risk 2. Genome wide association (GWA) studies have yielded multiple common sequence variants associated with prostate cancer risk. To date, more than 70 prostate cancer susceptibility loci have been identified, collectively explaining about 30% of the familial risk for the disease 3.

The great majority of GWA studies of prostate cancer have been performed in PSA‐screened populations (Western Europe and USA) and little is known about the risk estimates for populations from Central and Eastern Europe. An example is Romania were screening for prostate cancer is not common. The Romanian unscreened population represents a good opportunity to study inherited risk genetic variants since the over diagnosis of indolent forms is not yet a major problem. However, a comprehensive analysis of single nucleotide polymorphisms (SNPs) known to be associated with prostate cancer risk, based on GWA studies, has not been conducted in the Romanian population. Here, we report our replication efforts in the Romanian population for potential association of 34 literature‐reported SNPs with prostate cancer. We also examined whether any SNP was differentially associated with tumour grade or stage at diagnosis, with disease aggressiveness, and with the levels of PSA.

## Material and methods

### Cases and controls

Subjects included in this study were hospital patients admitted between 2008 and 2012 in two clinics in Bucharest (Urology Clinic ‘Th. Burghele’ and General Surgery Clinic ‘St. Mary’) for various urological and surgical conditions. We defined cases as men presenting for urinary tract symptoms suggesting prostatic hyperplasia, with positive digital rectal examination, abnormal PSA levels and first positive biopsy. Controls were patients admitted for urological and surgical conditions, excluding cancer. PSA level in plasma was measured for all subjects at hospital admission but was not used as an exclusion criteria. Each eligible subject gave written informed consent prior to enrolment and accepted the use of personal and clinical data and biological samples for genetic research. Trained interviewers performed face‐to‐face interview, using standardized questionnaires, to collect personal data (ethnicity, marital status, education, height and weight), lifestyle data (occupation, smoking, coffee and tea consumption) and medical history (personal and familial). DNA was extracted from whole blood at deCODE Genetics laboratories (Reykjavik, Iceland) for genotyping. The Bioethical Committee of the Romanian College of Physicians approved the study.

We staged the cases using UICC – TNM staging system 4. Men with prostate cancer staged I‐II cTNM were considered as low‐stage and III‐IV cTNM as high‐stage. Gleason score used in the study was based on transrectal ultrasound guided needle core biopsy. The same pathologist at The Urology Clinic ‘Th. Burghele’ reviewed all biopsy samples. Biopsies with Gleason score 1–7 were considered to be low‐grade and biopsies with Gleason 8–10 were considered to be high‐grade.

We defined aggressiveness according to pre‐treatment risk stratification used by Memorial Sloan‐Kettering and Seattle groups 5. For the analysis, we grouped low and intermediate risk cases as less‐aggressive group.

### Genotyping

We selected a panel of 34 SNPs found in large GWA studies 6, 7, 8, 9, 10, 11, 12, 13, 14, 15, 16, 17, 18, 19, 20, 21. The 34 SNPs were selected to represent the first prostate cancer risk loci reported to reach genome‐wide significance in GWA studies according to the Catalog of Published Genome‐Wide Association Studies (http://www.genome.gov/26525384). Genomic DNA extraction from peripheral blood was done using a semi‐automated platform for high quality, high throughput DNA extraction. The kits (Chemagic DNA Blood10k kit), the equipment (Chemagic Magnetic Separation Module MSM I) and the methods were from Chemagen (PerkinElmer chemagen Technologie GmbH, Baesweiler, Germany). Single SNP genotyping of the SNPs reported here was carried out by deCODE Genetics in Reykjavik, Iceland, applying the Centaurus (Epoch Biosciences, WA, USA) platform 22. The quality of each Centaurus SNP assay was evaluated by genotyping each assay in the CEU and/or YRI HapMap samples and comparing the results with the HapMap publicly released data. Assays with >1.5% mismatch rate were not used.

### Statistical analysis

We tested the Hardy**–**Weinberg equilibrium (HWE) for all 34 SNPs separately among cases and controls through Chi square test with one grade of freedom.

Allelic frequencies of SNPs in cases and controls were calculated and tested through Fisher's exact test. We defined the risk alleles as variants that corresponds to the disease *i.e*. cancer odds ratio (OR) greater than one. The association of each SNP with PSA levels was tested using a linear regression model of the PSA values, on the natural log scale, *versus* two independent variables: the number of minor alleles (taken as reference) and age, respectively.

All reported *P*‐values are uncorrected for multiple testing and are based on a two‐sided test. A level of *P* ≤ 0.05 was used to indicate statistical significance. Statistical analysis was performed using PLINK v1.07 (Center for Human Genetic Research, Boston, Massachusetts, United States and Broad Institute of Harvard & MIT, Cambridge, Massachusetts, United States; http://pngu.mgh.harvard.edu/purcell/plink/), R v3.2.0 (R Foundation for Statistical Computing, Vienna, Austria; http://www.R-project.org/) and Stata MP13 software (StataCorp LP, College Station, Texas, United States).

## Results

We genotyped 979 cases and 1027 controls, enrolled between May 2008 and Sept 2012. All recruited subjects were Romanian Caucasians. Table S1 presents the clinical characteristics of the cohort. 70.2% of cases had PSA levels >10 ng/ml reflecting an unscreened population, with significant clinical disease at presentation (77% with locally advanced tumours staged T3 and T4).

The genotyped SNPs did not deviate from HWE. MAFs (minor allele frequencies) of the effect alleles in controls did not differ from the original studies.

Single‐locus tests of association (cases *versus* controls) were conducted for each previously reported SNP. We calculated allelic ORs and 95% confidence intervals (CIs) and we compared the results with reference ORs from original GWAS (Table 1). Nineteen SNPs of 34 SNPs tested were nominally significantly (*P* < 0.05) associated with the disease. The association was in the same direction as in the reference studies with the magnitude of risk similar or greater than previously reported for variants on 4q24, 6q25.3, 7p15.2, 8q24.21, 11p15.5, 11q13.2. Five other SNPs on 2p21, 2p15, 4q22.3, 8p21.2, 17q12 showed direction of effect consistent with the original reports, but non‐significant. For the variants tested on 2q31.1, 3p12.1, 3q21.3, 7q21.3, 17p12, 17q24.3, 19q13.2 and 22q13.1, we could not reproduce the effect on risk for any of the disease phenotypes investigated. Based on *P*‐values, the strongest association observed was for rs445114 on 8q24.21 (*P* = 0.000013). The highest OR was 1.58 (for rs16901979 on 8q24.21).

**Table 1:** Association of 34 selected SNPs with prostate cancer risk

Locus	SNP	Romanian population							Reference studies			
		Risk allele	Cases	RAF cases	Controls	RAF controls	OR (95% CI)	*P* a value	Reported RAF	Reported per allele OR	Mapped gene	Reference
2p21	rs1465618	A	974	0.26	1019	0.23	1.15 (0.99–1.33)	0.069	0.23	1.08 (1.03–1.12)	Intronic *THADA*	Eeles 2009
2p15	rs721048	A	956	0.16	1014	0.14	1.13 (0.95–1.36)	0.164	0.19	1.15 (1.10–1.21)	Intronic in *EHBP1*	Gudmundsson 2008
2q31.1	rs12621278	A	977	0.97	1020	0.97	1.06 (0.72–1.56)	0.778	0.94	1.33 (1.25–1.43)	Intronic in *ITGA6*	Eeles 2009
3p12.1	rs2660753	C	953	0.83	1020	0.82	1.08 (0.91–1.27)	0.401	0.11	1.18 (1.06–1.31)	Intergenic	Eeles 2008
3q21.3	rs10934853	A	968	0.30	1020	0.29	1.04 (0.91–1.20)	0.554	0.28	1.12 (1.08–1.16)	Intergenic *EEFSEC*	Gudmundsson 2009
4q22.3	rs12500426	C	963	0.49	1021	0.47	1.08 (0.95–1.22)	0.240	0.46	1.08 1.05–1.12)	Intronic in *PDLIM5*	Eeles 2009
4q22.3	rs17021918	C	967	0.66	1013	0.65	1.02 (0.89–1.16)	0.789	0.65	1.11 (1.08–1.15)	*Intronic in PDLIM5*	Eeles 2009
4q24	rs7679673	C	969	0.56	1012	0.52	1.20 (1.06–1.37)	0.0037	0.55	1.1 (1.06–1.14)	*TET2*	Eeles 2009
5p15.33	rs2736098	G	943	0.72	1006	0.69	1.15 (1.00–1.33)	0.045	0.26	1.13 (1.06–1.21)	*TERT*	Rafnar 2009
5p15.33	rs401681	T	973	0.41	1016	0.40	1.08 (0.95–1.23)	0.258	0.55	1.07 (1.03–1.11)	*CLPTM1L*	Rafnar 2009
6q25.3	rs9364554	T	946	0.27	999	0.21	1.35 (1.16–1.57)	0.000076	0.29	1.17 (1.08–1.26)	Intronic in *SLC22A3*	Eeles 2008
7p15.2	rs10486567	G	962	0.81	1015	0.77	1.32 (1.12–1.54)	0.00046	0.77	1.12 (1.02–1.25)	Intronic in *JAZF1*	Thomas 2008
7q21.3	rs6465657	C	957	0.43	1019	0.42	1.03 (0.91–1.17)	0.652	0.46	1.12 (1.05–1.20)	Intronic in *LMTK2*	Eeles 2008
8p21.2	rs1512268	A	969	0.48	1014	0.45	1.13 (1.00–1.28)	0.056	0.45	1.18 (1.14–1.22)	*NKX3.1*	Eeles 2009
8q24.21	rs16901979	A	955	0.04	1007	0.03	1.58 (1.11–2.27)	0.0098	0.03	1.79 (1.36–2.34)	Intergenic *POU5F1B*	Gudmundsson 2007
8q24.21	rs16902094	G	949	0.17	1007	0.14	1.28 (1.07–1.52)	0.0056	0.15	1.21 (1.15–1.26)	Intergenic *POU5F1B*	Gudmundsson 2009
8q24.21	rs445114	T	950	0.66	1006	0.59	1.33 (1.18–1.52)	0.000013	0.64	1.14 (1.10–1.19)	Intergenic *POU5F1B*	Gudmundsson 2009
8q24.21	rs6983267	G	955	0.54	1014	0.51	1.15 (1.01–1.30)	0.032	0.50	1.26 (1.13–1.41)	Intergenic *POU5F1B*	Yeager 2007
8q24.21	rs1447295	A	968	0.12	1009	0.09	1.27 (1.03–1.57)	0.025	0.09	1.62 (1.43–1.77)	Intergenic *CASC8*	Gudmundsson 2007
10q11.23	rs10933994	T	964	0.45	996	0.39	1.28 (1.13–1.46)	0.00015	0.40	1.25 (1.17–1.34)	Promoter of *MSMB*	Eeles 2008. Thomas 2008
10q26.13	rs4962416	C	955	0.32	996	0.28	1.19 (1.03–1.36)	0.016	0.27	1.17 (1.05–1.30)	Intronic in *CTBP2*	Thomas 2008
11p15.5	rs7127900	A	968	0.22	1019	0.18	1.30 (1.11–1.52)	0.0011	0.20	1.22 (1.17–1.27)	TH ‐ *ASCL2*	Eeles 2009
11q13.2	rs12418451	A	939	0.35	976	0.30	1.22 (1.06–1.40)	0.0047	0.28	NR		Zheng 2009
11q13.3	rs11228565	A	961	0.23	1004	0.20	1.18 (1.02–1.38)	0.030	0.20	1.23 (1.16–1.31)	*Intergenic MIR3164 ‐ MYEOV*	Gudmundsson 2010
11q13.3	rs10896450	G	969	0.54	1011	0.47	1.30 (1.14–1.47)	0.000047	0.46	1.13 (1.06–1.21)		Gudmundsson 2009
17p12	rs4054823	T	964	0.54	1016	0.52	1.09 (0.95–1.23)	0.226	0.54^b^	1.13 (1.08–1.19)^b^		Xu 2010
17q12	rs11649743	G	969	0.84	1014	0.81	1.16 (0.89–1.37)	0.080	0.80	1.28 (1.07–1.52)	Intronic in *HNF1B*	Sun 2008
17q12	rs4430796	A	972	0.57	1017	0.52	1.22 (1.08–1.39)	0.0022	0.49	1.22 (1.15–1.30)	Intronic in *HNF1B*	Gudmundsson 2007
17q24.3	rs1859962	G	972	0.52	1017	0.50	1.08 (0.95–1.22)	0.253	0.46	1.20 (1.14–1.27)	Intergenic *CASC17*	Gudmundsson 2007
19q13.2	rs8102476	C	952	0.61	993	0.60	1.06 (0.93–1.20)	0.393	0.54	1.12 (1.08–1.15)	Intergenic	Gudmundsson 2009
19q13.33	rs2735839	G	965	0.88	1003	0.85	1.23 (1.02–1.49)	0.026	0.85	1.2 (1.10–1.33)	*KLK3 ⁄ KLK3*	Eeles 2008
22q13.1	rs9623117	C	879	0.23	987	0.23	1.00 (0.85–1.17)	1.0	0.21	1.18 (1.11–1.26)	Intronic *TNRC6B*	Sun 2008
22q13.2	rs5759167	G	918	0.57	933	0.53	1.16 (1.02–1.33)	0.019	NR	1.16 (1.14–1.20)		Eeles 2009
Xp11.22	rs5945572	A	916	0.41	1020	0.36	1.24 (1.03–1.49)	0.024	0.35	1.23 (1.16–1.30)	*NUDT11*	Gudmundsson 2008

a *P*‐value uncorrected for multiple testing.

b In aggressive *versus* non‐aggressive cases.

RAF: Risk allele frequency.

Secondly, we tested if these 34 SNPs are differentially associated with clinico‐pathological characteristics of the disease at diagnosis: clinical stage (cTNM), Gleason score on first biopsy, pre‐operatory PSA levels and aggressiveness.

High stage cTNM did not modify the risk estimates. The association with the risk alleles at 6q25.3 (rs9364554) and 11q13.3 (rs10896450) became stronger for cases staged cTNM I and II (Table S2). Rs2735839 (on 19q13.33) was strongly associated only with the low stage cTNM (OR = 1.69, CI = 1.11–2.63, *P* = 0.009).

In the analysis of the pathological features of the tumours based on Gleason grade on biopsy, we found a significant increased risk for high grade tumours (Gleason 8–10) associated with the variants on 8q24.21 (Table S2).

The ORs for prostate cancer did not differ significantly by perioperative PSA levels (Table S3). However, the risk associated with rs401681 on 5p15.33 and rs11649743 on 17q12 became significant stronger (OR = 1.19, CI = 1.02–1.39, *P* = 0.027 and OR = 1.23, CI = 1.00–1.52, *P* = 0.047 respectively) for high PSA values.

A quantitative regression association of the PSA values with the 34 SNP's was performed in cases, controls, and in the mixed group (Table S4). However, given the correlation (or confounding) of the high PSA levels with the disease, the most relevant analysis is the one for the population controls only. This analysis detected four SNPs with low p‐values: rs2735839 (19q13.33, *P* = 0.0004), rs2736098 (5p15.33, *P* = 0.023), rs10993994 (10q11.23, *P* = 0.010), and rs4962416 (10q26.13, *P* = 0.018). The first three SNPs were previously reported in association with high PSA levels in other populations 23 and therefore can be considered only indirectly associated with prostate cancer. Gudmundsson *et al*. also reported another SNP associated with PSA, rs10788160 (10q26.12, close to FGFR2 gene), whereas our fourth SNP (rs4962416) is located 3.66 Mb further, within the gene CTBP2, and not in LD with rs10788160. Rs4962416 has been previously associated with prostate cancer, but not with the PSA levels in non‐cancer patients. The increase of PSA levels corresponds to 12% for each copy of the minor allele C (or 0.113 on the log scale). The strongest association with PSA was for rs2735839, which is located near the KLK3 gene that encodes PSA, with 29% increase for each copy of the major allele G, consistent with previous results reported by Gudmundsson *et al*.

When cases were divided into categories of disease severity by a combination of high‐risk clinical variables (cTNM, Gleason score, PSA levels at diagnosis), three SNPs showed significant association but only with less aggressive disease (rs1465618, rs721048, rs17021918). The risk at 6q25.3, 11q13.3 and 19q13.33 (rs2735839) was significantly higher for the cases having less severe disease (Table S4).

Markers on 6q25.3 (rs9364554) and on 11q13.3 (rs10896450) associated with all the phenotypes tested with ORs particularly high for the less aggressive variants.

## Discussion

In recent years, a large amount of information on prostate cancer risk associated SNPs are available for multiple white case**–**control samples. However, less is known about whether these associations can be consistently replicated in Eastern European populations 24. A recent study reported on the associations of prostate cancer risk with two loci on chromosome 17q12 (rs3760511 and rs7501939) in the Serbian population 25. Another large study on Polish men reported on the positive associations with prostate cancer for 5 of 11 studied SNPs 26.

To our best knowledge, this is the first study set out to examine 34 SNPs previously identified for association with prostate cancer in a Romanian hospital‐based case**–**control series of unscreened men and to evaluate if there is any variation in risk by diseases severity at diagnosis.

We found evidence of similar or higher association with prostate cancer risk for 24 SNPs investigated. Six other literature‐reported variants reached nominal significance only for particular forms of the disease. The previously reported associations with PrCa for loci on 2q31.1, 3p12.1, 3q21.3, 7q21.3, 17p12, 17q24.3, 19q13.2, 22q13.1 were not replicated in our study. Our failure to replicate all markers is not unexpected as previous studies report only 25–60% successful replication of prostate cancer association SNPs 27. This failure can possibly be linked to the sample size leading to insufficient statistical power, but more likely to the much weaker, if not even inexistent, effect in the Romanian population, and hence to a higher, population dependent, cancer heterogeneity 28.

In our study population, the strongest nominal associations were for variants on 8q24; for any SNPs that were significant in the allelic analysis, the OR was consistently more extreme for high‐grade prostate cancer than reported in discovery studies 29. However, the results for the aggressive phenotype were similar to the results of all prostate cancer cases indicating that SNPs on 8q24 do not offer substantial discrimination between these two distinguishable phenotypes.

We observed a strong association with less aggressive disease for rs1465618 on 2p21 (OR = 1.37; CI = 1.06–1.76, *P* = 0.016) and rs721048 on 2p15 (OR = 1.43; CI = 1.05–1.93, *P* = 0.019) which is in contrast with other previous reports 6, 30. The risk allele at rs17021918 was associated only with low aggressiveness; similar findings were reported by Shui 31. For the SNPs associated with PSA levels in controls, we observed a stronger association with low‐grade tumours (rs10993994 and rs4962416 on 10q, rs2735839 on 19q13.33) or high‐grade tumours (rs2736098 on 5p15.33).

Previous reports have shown genomic variants at 10q to be associated with both PSA levels in healthy controls and with cancer risk in average and high‐risk Caucasian men 32, 33, 34. The role of rs10993994 in the discrimination of aggressive *versus* non‐aggressive cancers is controversial since the chromosome 10q11 prostate cancer risk locus is associated with decreased levels of MSMB and increased levels of NCOA4 RNA expression; both genes have been suggested to mediate prostate tumourigenesis (initiation and progression) 35.

Our results for rs2735839, rs2736098, and rs10993994 show a clear tendency for the alleles associated with prostate cancer risk to be also associated with PSA levels in controls which is in line with results reported by Gudmundsson *et al*. 23. In addition we also observed a similar effect for rs4962416, within the gene CTBP2, suggesting that this gene may also be primarily linked to PSA, and not directly to cancer. Since the obtained *P*‐value is not convincingly low, considering multiple testing corrections, this association needs further confirmation. The purpose of this study was a replication of previously known SNPs, and therefore the *P*‐values were not corrected for multiple testing. On the other hand, a limitation is that the sample size was smaller than in the previous studies, and our risk estimates have larger confidence intervals.

Our present cohort of about 2000 individuals has a reasonable power to detect common variants with allelic relative risk of about 1.2 or larger. To evaluate the possible effect of the unconfirmed SNPs or to detect novel risk variants in the Romanian population further larger samples are needed.

## Conclusions

Our study provides evidence that the majority of previously validated prostate cancer SNPs associates with risk in the Romanian non‐screened population, having relevant clinical disease, and can be considered for inclusion in future risk models of potential clinical utility.

## Conflicts of interest

The authors confirm that there are no conflicts of interest.

## Supporting information


**Table S1** Characteristics of controls and cancer cases.
**Table S2** Association of the 34 SNPs with clinical TNM stage and Gleason score on biopsy.
**Table S3** Association of the 34 SNPs with PSA levels and disease aggressiveness.
**Table S4** Association of the 34 SNPs with PSA using linear regression.
