# Supplementary material for: Replication study of 34 common SNPs associated with prostate cancer in the Romanian population
Source: J Cell Mol Med. 2016 Jan 15;20(4):594–600. doi: 10.1111/jcmm.12729 (PMC5126261; doi:10.1111/jcmm.12729)
Supplement: Supplementary file 1 — Table S1 Characteristics of controls and cancer cases. Table S2 Association of the 34 SNPs with clinical TNM stage and Gleason score on biopsy. Table S3 Association of the 34 SNPs with PSA levels and disease aggressiveness. Table S4 Association of the 34 SNPs with PSA using linear regression. [file JCMM-20-594-s001.docx]

Supp. Table 1: Characteristics of controls and cancer cases

| Characteristics | Controls | Cases |
| --- | --- | --- |
| N (patients) | 1027 | 979 |
| Age (years) |  |  |
| Mean ± SD | 63.2 ± 12.8 | 70.9 ± 8.0 |
| Range | 19 - 92 | 44 - 92 |
| Median | 65 | 71 |
| Missing, N (%) | 1 (0.1) | - |
|  |  |  |
| Age of onset (years) |  |  |
| Mean ± SD |  | 70.3 ± 8.0 |
| Range |  | 39 - 92 |
| Median |  | 71 |
| Missing, N (%) |  | 6 (6.0) |
|  |  |  |
| Family history of cancer, N (%) | 101 (9.8) | 172 (17.6) |
| Missing, N (%) | - | - |
|  |  |  |
| Family history of prostate cancer, N (%) | 23 (2.2) | 40 (4.1) |
| Missing, N (%) | - | - |
|  |  |  |
| PSA (ng/ml) |  |  |
| Mean ± SD | 3.0 ± 6.1 | 63.9 ± 172.8 |
| Range | 0.001 - 143.8 | 0.01 - 2000 |
| Median | 1.3 | 21 |
| Missing, N (%) | 23 (2.2) | 6 (6.0) |
|  |  |  |
| PSA, N (%) |  |  |
| < 2.5 ng/ml | 693 (67.5) | 37 (3.8) |
| 2.5 ng/ml - 4.5 ng/ml | 115 (11.2) | 26 (2.7) |
| 4.5 ng/ml - 10 ng/ml | 140 (13.6) | 221 (22.6) |
| > 10 ng/ml | 56 (5.5) | 689 (70.4) |
| Missing, N (%) | 23 (2.2) | 6 (0.6) |
|  |  |  |
| Gleason score, N (%) |  |  |
| ≤ 6 |  | 178 (18.2) |
| 7 |  | 442 (45.2) |
| ≥ 8 |  | 342 (34.9) |
| Missing, N (%) |  | 17 (1.7) |
|  |  |  |
| Clinical T stage, N (%) |  |  |
| T1A |  | 21 (2.2) |
| T1B + T1C |  | 162 (16.6) |
| T2 |  | 72 (7.4) |
| T3 |  | 488 (49.9) |
| T4 |  | 228 (23.3) |
| Missing, N (%) |  | 8 (0.8) |
|  |  |  |
| Clinical nodal stage, N (%) |  |  |
| N0 |  | 205 (20.9) |
| N1 |  | 30 (3.1) |
| Nx |  | 736 (75.2) |
| Missing, N (%) |  | 8 (0.8) |
|  |  |  |
| Clinical metastasis stage N (%) |  |  |
| M0 |  | 209 (21.4) |
| M1 |  | 97 (9.9) |
| Mx |  | 665 (67.9) |
| Missing, N (%) |  | 8 (0.8) |
|  |  |  |
| Aggressiveness |  |  |
| Risk groupings according to Memorial Sloan-Kettering and Seattle groups |  |  |
| Low risk |  | 67 (6.8) |
| (Clinical stage ≤T2a, Gleason score ≤6, PSA<10ng/ml), N (%) |  |  |
| Intermediate risk |  | 117 (12) |
| (One elevated risk factor: Clinical stage >T2a, Gleason score ≥7, PSA≥10ng/ml), N (%) |  |  |
| High risk |  | 774 (79.1) |
| (Two elevated risk factors), N (%) |  |  |
| Missing, N (%) |  | 21 (2.2) |
| PSA = prostate specific antigen; SD= standard deviation |  |  |

Supp. Table 2: Association of the 34 SNPs with clinical TNM stage and Gleason score on biopsy

| SNP-risk allele | Clinical TNM stage I+II  cases vs. controls | | Clinical TNM stage III+IV cases vs. controls | | Gleason score 1-7 cases vs. controls | | Gleason score 8-10 cases vs. controls | |
| --- | --- | --- | --- | --- | --- | --- | --- | --- |
|  | OR (95% CI) | P | OR (95% CI) | P | OR (95% CI) | P | OR (95% CI) | P |
| rs1465618-A | 1.06 ( 0.79, 1.42) | 7.1E-01 | 1.16 ( 0.99, 1.35) | 6.2E-02 | 1.15 ( 0.97, 1.36) | 1.1E-01 | 1.15 ( 0.94, 1.41) | 1.8E-01 |
| rs721048-A | 1.23 ( 0.86, 1.72) | 2.5E-01 | 1.12 ( 0.92, 1.35) | 2.6E-01 | 1.14 ( 0.93, 1.40) | 2.0E-01 | 1.09 ( 0.84, 1.40) | 5.2E-01 |
| rs12621278-A | 1.30 ( 0.58, 3.45) | 7.1E-01 | 1.02 ( 0.68, 1.54) | 1.0E+00 | 0.90 ( 0.59, 1.39) | 6.1E-01 | 1.47 (0.81, 2.86) | 2.2E-01 |
| rs2660753-C | 1.30 ( 0.92, 1.89) | 1.4E-01 | 1.04 (0.88, 1.25) | 6.6E-01 | 1.12 ( 0.93, 1.37) | 2.3E-01 | 0.95 (0.76, 1.20) | 7.3E-01 |
| rs10934853-A | 1.00 ( 0.75, 1.31) | 1.0E+00 | 1.05 ( 0.90, 1.21) | 5.3E-01 | 1.02 ( 0.87, 1.19) | 8.7E-01 | 1.10 ( 0.90, 1.33) | 3.5E-01 |
| rs12500426-C | 0.96 ( 0.75, 1.24) | 8.0E-01 | 1.10 ( 0.97, 1.26) | 1.4E-01 | 1.06 ( 0.92, 1.22) | 4.5E-01 | 1.11 ( 0.93, 1.32) | 2.7E-01 |
| rs17021918-C | 1.28 ( 0.97, 1.69) | 7.8E-02 | 0.98 ( 0.85, 1.14) | 8.3E-01 | 1.09 ( 0.93, 1.27) | 3.0E-01 | 0.93 ( 0.78, 1.12) | 4.8E-01 |
| rs7679673-C | 1.15( 0.90, 1.49) | 2.7E-01 | 1.12( 1.06, 1.39) | 3.6E-03 | 1.18 ( 1.02, 1.35) | 2.9E-02 | 1.28 ( 1.06, 1.54) | 6.7E-03 |
| rs2736098-G | 1.20 ( 0.91, 1.61) | 1.9E-01 | 1.14 ( 0.98, 1.32) | 7.7E-02 | 1.05 ( 0.90, 1.23) | 5.2E-01 | 1.35 ( 1.11, 1.67) | 3.0E-03 |
| rs401681-T | 1.09 ( 0.85, 1.40) | 4.9E-01 | 1.08 ( 0.94, 1.24) | 2.6E-01 | 1.03 ( 0.89, 1.19) | 7.4E-01 | 1.16 ( 0.97, 1.39) | 9.5E-02 |
| rs9364554-T | 1.49 ( 1.12, 1.98) | 5.1E-03 | 1.32 ( 1.13, 1.55) | 4.7E-04 | 1.39 ( 1.17, 1.65) | 1.2E-04 | 1.27 ( 1.02, 1.56) | 2.7E-02 |
| rs10486567-G | 1.28 ( 0.93, 1.75) | 1.2E-01 | 1.33 (1.14, 1.56) | 5.3E-04 | 1.30 ( 1.09, 1.56) | 3.7E-03 | 1.33 ( 1.06, 1.67) | 1.0E-02 |
| rs6465657-C | 0.94 ( 0.73, 1.21) | 6.6E-01 | 1.05 ( 0.92, 1.20) | 4.6E-01 | 1.06 ( 0.91, 1.22) | 4.6E-01 | 0.99 ( 0.83, 1.19) | 9.6E-01 |
| rs1512268-A | 1.27 ( 0.99, 1.64) | 5.4E-02 | 1.11 ( 0.97, 1.26) | 1.3E-01 | 1.26 ( 1.09, 1.46) | 1.3E-03 | 0.92 ( 0.77, 1.10) | 4.0E-01 |
| rs16901979-A | 1.44 ( 0.69, 2.76) | 2.7E-01 | 1.62 ( 1.12, 2.36) | 8.6E-03 | 1.39 ( 0.92, 2.10) | 1.0E-01 | 1.89 ( 1.19, 2.96) | 4.6E-03 |
| rs16902094-G | 1.26 ( 0.88, 1.77) | 1.8E-01 | 1.28 ( 1.07, 1.54) | 7.3E-03 | 1.23 ( 1.00, 1.50) | 4.7E-02 | 1.37 ( 1.08, 1.74) | 8.8E-03 |
| rs445114-T | 1.20 ( 0.93, 1.56) | 1.4E-01 | 1.37 ( 1.19, 1.56) | 8.0E-06 | 1.27 ( 1.09, 1.47) | 1.7E-03 | 1.49 ( 1.23, 1.82) | 2.6E-05 |
| rs6983267-G | 0.93 ( 0.72, 1.19) | 5.4E-01 | 1.20 ( 1.05, 1.37) | 6.6E-03 | 1.09 ( 0.93, 1.25) | 2.8E-01 | 1.28 ( 1.08, 1.54) | 5.5E-03 |
| rs1447295-A | 1.14 ( 0.74, 1.71) | 5.2E-01 | 1.28 ( 1.03, 1.59) | 2.8E-02 | 1.20 ( 0.94, 1.53) | 1.3E-01 | 1.36 ( 1.02, 1.80) | 3.2E-02 |
| rs10993994-T | 1.21 ( 0.94, 1.56) | 1.3E-01 | 1.30 ( 1.14, 1.49) | 1.1E-04 | 1.36 ( 1.17, 1.57) | 3.6E-05 | 1.17 ( 0.97, 1.40) | 9.1E-02 |
| rs4962416-C | 1.20 ( 0.91, 1.57) | 1.9E-01 | 1.19 ( 1.03, 1.38) | 1.7E-02 | 1.21 ( 1.03, 1.42) | 1.9E-02 | 1.19 ( 0.98, 1.44) | 7.0E-02 |
| rs7127900-A | 1.20 ( 0.87, 1.63) | 2.6E-01 | 1.32 ( 1.12, 1.56) | 8.7E-04 | 1.26 ( 1.05, 1.50) | 1.2E-02 | 1.39 ( 1.12, 1.72) | 2.9E-03 |
| rs12418451-A | 1.23 ( 0.93, 1.61) | 1.3E-01 | 1.22 ( 1.05, 1.41) | 6.7E-03 | 1.25 ( 1.07, 1.46) | 4.1E-03 | 1.18 ( 0.97, 1.43) | 8.9E-02 |
| rs11228565-A | 0.97 ( 0.70, 1.32) | 8.8E-01 | 1.22 ( 1.04, 1.43) | 1.5E-02 | 1.19 ( 0.99, 1.41) | 5.7E-02 | 1.21 ( 0.98, 1.50) | 8.1E-02 |
| rs10896450-G | 1.47 ( 1.14, 1.89) | 2.4E-03 | 1.26 ( 1.11, 1.44) | 5.2E-04 | 1.33 ( 1.15, 1.54) | 9.2E-05 | 1.27 ( 1.06, 1.52) | 7.7E-03 |
| rs4054823-T | 0.79 ( 0.63, 1.03) | 9.1E-02 | 1.04 ( 0.93, 1.30) | 6.2E-02 | 1.08 ( 0.93, 1.23) | 3.6E-01 | 1.08 ( 0.90, 1.28) | 4.0E-01 |
| rs11649743-G | 1.11 ( 0.80, 1.56) | 5.8E-01 | 1.18 ( 0.89, 1.41) | 7.3E-02 | 1.18 ( 0.97, 1.43) | 8.8E-02 | 1.11 ( 0.88, 1.41) | 3.9E-01 |
| rs4430796-A | 1.12 ( 0.88, 1.45) | 3.5E-01 | 1.23 ( 1.08, 1.41) | 2.1E-03 | 1.18 ( 1.02, 1.37) | 2.5E-02 | 1.23 ( 1.03, 1.47) | 1.9E-02 |
| rs1859962-G | 0.92 ( 0.71, 1.18) | 5.0E-01 | 1.10 ( 0.96, 1.26) | 1.5E-01 | 1.12 ( 0.97, 1.29) | 1.3E-01 | 1.01 ( 0.85, 1.21) | 8.9E-01 |
| rs8102476-C | 1.12 ( 0.86, 1.45) | 4.1E-01 | 1.05 ( 0.92, 1.22) | 4.3E-01 | 1.04 ( 0.89, 1.20) | 6.0E-01 | 1.09 ( 1.30, 0.90) | 3.8E-01 |
| rs2735839-G | 1.69 ( 1.11, 2.63) | 9.8E-03 | 1.16 ( 0.96, 1.42) | 1.1E-01 | 1.14 ( 1.14, 1.19) | 1.7E-03 | 1.03 ( 0.81, 1.33) | 8.5E-01 |
| rs9623117-C | 0.83 ( 0.59, 1.14) | 2.8E-01 | 1.04 ( 0.88, 1.22) | 6.8E-01 | 1.10 ( 0.92, 1.31) | 2.9E-01 | 0.84 ( 0.67, 1.06) | 1.3E-01 |
| rs5759167-G | 1.18 ( 0.91, 1.54) | 2.2E-01 | 1.18 ( 1.02, 1.35) | 2.2E-02 | 1.18 (1.01, 1.37) | 3.6E-02 | 1.16 ( 0.96, 1.41) | 1.1E-01 |
| rs5945572-A | 1.10 ( 0.76, 1.59) | 5.8E-01 | 1.28 ( 1.05, 1.56) | 1.4E-02 | 1.29 ( 1.04, 1.60) | 1.8E-02 | 1.13 ( 0.86, 1.47) | 3.9E-01 |

Supp. Table 3: Association of the 34 SNPs with PSA levels and disease aggressiveness

| SNP-risk allele | PSA < 20 ng/ml  cases vs. controls | | PSA ≥ 20 ng/ml  cases vs. controls | | Less aggressive disease  cases vs. controls | | Aggressive disease  cases vs. controls | |
| --- | --- | --- | --- | --- | --- | --- | --- | --- |
|  | OR (95% CI) | P | OR (95% CI) | P | OR (95% CI) | P | OR (95% CI) | P |
| rs1465618-A | 1.14 ( 0.95, 1.37) | 1.5E-01 | 1.14 ( 0.95, 1.36) | 1.6E-01 | 1.37 ( 1.06, 1.76) | 1.6E-02 | 1.09 ( 0.93, 1.27) | 3.0E-01 |
| rs721048-A | 1.17 ( 0.94, 1.46) | 1.6E-01 | 1.09 ( 0.87, 1.36) | 4.4E-01 | 1.43 ( 1.05, 1.93) | 1.9E-02 | 1.07 ( 0.88, 1.29) | 5.3E-01 |
| rs12621278-A | 1.19 ( 0.72, 2.00) | 5.5E-01 | 0.96 ( 0.61, 1.54) | 8.2E-01 | 0.92 (0.48, 1.89) | 7.4E-01 | 1.09 ( 0.72, 1.67) | 6.9E-01 |
| rs2660753-C | 1.18 ( 0.95, 1.47) | 1.4E-01 | 0.99 ( 0.81, 1.22) | 9.6E-01 | 1.28 ( 0.93, 1.79) | 1.3E-01 | 1.03 ( 0.86, 1.23) | 7.6E-01 |
| rs10934853-A | 1.07 ( 0.90, 1.27) | 4.6E-01 | 1.02 ( 0.86, 1.21) | 8.0E-01 | 0.94 ( 0.73, 1.22) | 7.1E-01 | 1.07 ( 0.92, 1.24) | 3.7E-01 |
| rs12500426-C | 1.03 ( 0.88, 1.20) | 7.5E-01 | 1.13 ( 0.97, 1.32) | 1.2E-01 | 0.94 ( 0.75, 1.18) | 6.1E-01 | 1.12 ( 0.98, 1.28) | 1.0E-01 |
| rs17021918-C | 1.08 ( 0.91, 1.27) | 4.3E-01 | 0.98 ( 8.33, 1.15) | 8.1E-01 | 1.28 ( 1.00, 1.67) | 4.7E-02 | 0.97 ( 0.84, 1.12) | 7.0E-01 |
| rs7679673-C | 1.15 ( 0.98, 1.35) | 8.9E-02 | 1.28 ( 1.09, 1.49) | 1.9E-03 | 1.10 ( 0.88, 1.39) | 4.3E-01 | 1.23 ( 1.09, 1.43) | 1.5E-03 |
| rs2736098-G | 1.02 (0.85, 1.20) | 8.6E-01 | 1.30 ( 1.10, 1.56) | 2.8E-03 | 1.02 ( 0.79, 1.32) | 9.0E-01 | 1.19 ( 1.02, 1.39) | 2.2E-02 |
| rs401681-T | 0.97 ( 0.82, 1.14) | 7.2E-01 | 1.19 ( 1.02, 1.39) | 2.7E-02 | 0.96 ( 0.76, 1.21) | 7.3E-01 | 1.11 ( 0.97, 1.27) | 1.3E-01 |
| rs9364554-T | 1.37 ( 1.14, 1.65) | 8.7E-04 | 1.32 ( 1.10, 1.59) | 2.4E-03 | 1.50 ( 1.15, 1.94) | 2.2E-03 | 1.31 ( 1.12, 1.54) | 7.1E-04 |
| rs10486567-G | 1.41 ( 1.15, 1.72) | 5.6E-04 | 1.22 ( 1.01, 1.49) | 3.5E-02 | 1.39 ( 1.04, 1.89) | 2.5E-02 | 1.30 (1.10, 1.54) | 1.5E-03 |
| rs6465657-C | 1.00 ( 0.85, 1.17) | 1.0E+00 | 1.07 ( 0.91, 1.25) | 4.1E-01 | 1.01 ( 0.79, 1.27) | 1.0E+00 | 1.04 ( 0.91, 1.20) | 5.4E-01 |
| rs1512268-A | 1.19 ( 1.01, 1.39) | 3.2E-02 | 1.08 ( 0.92, 1.26) | 3.5E-01 | 1.21 ( 0.96, 1.52) | 1.1E-01 | 1.12 ( 0.98, 1.28) | 9.1E-02 |
| rs16901979-A | 1.51 ( 0.97, 2.33) | 5.7E-02 | 1.67 ( 1.09, 2.53) | 1.3E-02 | 1.60 ( 0.85, 2.86) | 1.3E-01 | 1.58 ( 1.08, 2.30) | 1.6E-02 |
| rs16902094-G | 1.29 ( 1.04, 1.60) | 2.2E-02 | 1.27 ( 1.02, 1.57) | 3.0E-02 | 1.22 ( 0.88, 1.67) | 2.2E-01 | 1.29 ( 1.07, 1.55) | 7.5E-03 |
| rs445114-T | 1.37 ( 1.16, 1.61) | 1.6E-04 | 1.32 ( 1.12, 1.54) | 7.4E-04 | 1.33 (1.04, 1.69) | 2.0E-02 | 1.33 ( 1.16, 1.54) | 3.4E-05 |
| rs6983267-G | 1.16 ( 0.99, 1.37) | 6.2E-02 | 1.14 ( 0.97, 1.33) | 1.0E-01 | 0.99 ( 0.78, 1.25) | 1.0E+00 | 1.19 ( 1.04, 1.37) | 8.2E-03 |
| rs1447295-A | 1.08 ( 0.82, 1.41) | 5.9E-01 | 1.45 ( 1.13, 1.86) | 2.8E-03 | 0.94 ( 0.62, 1.41) | 8.4E-01 | 1.33 ( 1.07, 1.66) | 1.1E-02 |
| rs10993994-T | 1.16 ( 0.99, 1.36) | 6.8E-02 | 1.41 ( 1.21, 1.65) | 1.4E-05 | 1.16 ( 0.92, 1.46) | 2.2E-01 | 1.32 ( 1.15, 1.51) | 5.7E-05 |
| rs4962416-C | 1.19 ( 1.00, 1.41) | 4.9E-02 | 1.19 ( 1.01, 1.41) | 4.0E-02 | 1.08 ( 0.84, 1.39) | 5.3E-01 | 1.22 ( 1.06, 1.42) | 6.8E-03 |
| rs7127900-A | 1.28 ( 1.05, 1.55) | 1.3E-02 | 1.33 ( 1.09, 1.61) | 3.8E-03 | 1.15 ( 0.86, 1.53) | 3.1E-01 | 1.34 ( 1.13, 1.58) | 5.5E-04 |
| rs12418451-A | 1.24 ( 1.05, 1.47) | 1.3E-02 | 1.19 ( 1.01, 1.41) | 3.8E-02 | 1.25 ( 0.97, 1.60) | 6.9E-02 | 1.21 ( 1.05, 1.40) | 8.3E-03 |
| rs11228565-A | 1.20 ( 0.99, 1.45) | 5.9E-02 | 1.16 ( 0.96, 1.40) | 1.2E-01 | 1.24 ( 0.94, 1.62) | 1.1E-01 | 1.17 ( 0.99, 1.37) | 6.3E-02 |
| rs10896450-G | 1.31 ( 1.12, 1.54) | 5.8E-04 | 1.28 ( 1.10, 1.50) | 1.5E-03 | 1.40 ( 1.11, 1.76) | 3.6E-03 | 1.27 ( 1.11, 1.45) | 4.5E-04 |
| rs4054823-T | 1.03 ( 0.88, 1.20) | 6.9E-01 | 1.11 ( 0.95, 1.30) | 1.7E-01 | 0.92 ( 0.97, 1.16) | 5.3E-01 | 1.11 (0.97, 1.27) | 1.2E-01 |
| rs11649743-G | 1.11 ( 0.90, 1.37) | 3.3E-01 | 1.23 ( 1.00, 1.52) | 4.7E-02 | 1.10 ( 0.81, 1.49) | 5.6E-01 | 1.18 (0.99, 1.14) | 6.9E-02 |
| rs4430796-A | 1.20 ( 1.02, 1.41) | 2.4E-02 | 1.23 ( 1.05, 1.43) | 8.2E-03 | 1.09 ( 0.86, 1.37) | 5.0E-01 | 1.25 ( 1.09, 1.43) | 1.3E-03 |
| rs1859962-G | 1.07 ( 0.91, 1.25) | 4.1E-01 | 1.08 ( 0.92, 1.26) | 3.5E-01 | 1.00 ( 0.79, 1.25) | 1.0E+00 | 1.10 ( 0.96, 1.25) | 1.8E-01 |
| rs8102476-C | 1.11 ( 0.94, 1.30) | 2.2E-01 | 1.03 ( 0.88, 1.20) | 7.5E-01 | 1.08 ( 0.85, 1.37) | 5.6E-01 | 1.06 ( 0.93, 1.22) | 3.8E-01 |
| rs2735839-G | 1.20 ( 0.96, 1.54) | 1.1E-01 | 1.25 ( 0.99, 1.59) | 5.8E-02 | 1.69 ( 1.15, 2.50) | 5.1E-03 | 1.16 ( 0.95, 1.41) | 1.3E-01 |
| rs9623117-C | 1.04 ( 0.85, 1.26) | 7.3E-01 | 0.98 ( 0.81, 1.19) | 8.5E-01 | 0.96 ( 0.71, 1.28) | 8.3E-01 | 1.01 ( 0.86, 1.20) | 9.0E-01 |
| rs5759167-G | 1.14 ( 0.96, 1.33) | 1.4E-01 | 1.20 ( 1.03, 1.43) | 2.0E-02 | 1.08 ( 0.85, 1.37) | 5.6E-01 | 1.19 ( 1.04, 1.37) | 1.0E-02 |
| rs5945572-A | 1.27 ( 1.00, 1.60) | 4.7E-02 | 1.23 ( 0.97, 1.55) | 8.1E-02 | 1.30 ( 0.92, 1.83) | 1.2E-01 | 1.23 ( 1.01, 1.50) | 4.0E-02 |

Supp. Table 4: Association of the 34 SNPs with PSA using linear regression

| SNP/minor allele | Cases | | | Controls | | | All | | |
| --- | --- | --- | --- | --- | --- | --- | --- | --- | --- |
|  | Coefficient | Standard Error | P | Coefficient | Standard Error | P | Coefficient | Standard Error | P |
| rs1465618/A | -0.028 | 0.077 | 7.14E-01 | 0.114 | 0.06 | 5.78E-02 | 0.129 | 0.066 | 5.05E-02 |
| rs721048/A | -0.071 | 0.092 | 4.40E-01 | -0.142 | 0.075 | 5.81E-02 | -0.037 | 0.081 | 6.47E-01 |
| rs12621278/G | 0.015 | 0.204 | 9.41E-01 | 0.026 | 0.155 | 8.65E-01 | 0.094 | 0.173 | 5.89E-01 |
| rs2660753/T | 0.131 | 0.091 | 1.52E-01 | 0.036 | 0.065 | 5.82E-01 | -0.005 | 0.075 | 9.42E-01 |
| rs10934853/A | -0.089 | 0.072 | 2.19E-01 | 0.091 | 0.057 | 1.10E-01 | 0.021 | 0.063 | 7.37E-01 |
| rs12500426/C | 0.128 | 0.065 | 5.01E-02 | -0.028 | 0.049 | 5.69E-01 | 0.054 | 0.055 | 3.32E-01 |
| rs17021918/T | 0.071 | 0.07 | 3.13E-01 | -0.054 | 0.052 | 2.95E-01 | -0.055 | 0.059 | 3.46E-01 |
| rs7679673/A | -0.076 | 0.067 | 2.59E-01 | 0.043 | 0.05 | 3.83E-01 | -0.12 | 0.056 | 3.32E-02 |
| rs2736098/A | -0.186 | 0.075 | 1.37E-02 | -0.118 | 0.052 | 2.28E-02 | -0.222 | 0.061 | 2.50E-04 |
| rs401681/T | 0.107 | 0.067 | 1.08E-01 | -0.037 | 0.052 | 4.76E-01 | 0.084 | 0.058 | 1.45E-01 |
| rs9364554/T | 0.046 | 0.076 | 5.47E-01 | 0.047 | 0.063 | 4.50E-01 | 0.198 | 0.067 | 3.12E-03 |
| rs10486567/A | 0.031 | 0.085 | 7.12E-01 | -0.079 | 0.061 | 1.94E-01 | -0.162 | 0.07 | 1.99E-02 |
| rs6465657/C | 0.037 | 0.067 | 5.87E-01 | 0.049 | 0.05 | 3.28E-01 | 0.027 | 0.057 | 6.35E-01 |
| rs1512268/A | -0.096 | 0.067 | 1.53E-01 | 0.065 | 0.05 | 1.89E-01 | 0.058 | 0.056 | 3.05E-01 |
| rs16901979/A | 0.148 | 0.162 | 3.62E-01 | 0.108 | 0.152 | 4.77E-01 | 0.352 | 0.151 | 1.97E-02 |
| rs16902094/G | 0.058 | 0.089 | 5.15E-01 | 0.004 | 0.072 | 9.58E-01 | 0.163 | 0.078 | 3.75E-02 |
| rs445114/C | -0.022 | 0.07 | 7.55E-01 | 0.017 | 0.051 | 7.34E-01 | -0.161 | 0.058 | 5.26E-03 |
| rs6983267/T | -0.019 | 0.065 | 7.68E-01 | -0.045 | 0.049 | 3.60E-01 | -0.078 | 0.055 | 1.55E-01 |
| rs1447295/A | 0.104 | 0.106 | 3.26E-01 | 0.02 | 0.088 | 8.22E-01 | 0.162 | 0.094 | 8.52E-02 |
| rs10993994/T | 0.117 | 0.067 | 8.23E-02 | 0.133 | 0.052 | 1.00E-02 | 0.268 | 0.057 | 3.00E-06 |
| rs4962416/C | 0.019 | 0.073 | 7.96E-01 | 0.133 | 0.056 | 1.78E-02 | 0.162 | 0.062 | 9.05E-03 |
| rs7127900/A | -0.072 | 0.082 | 3.80E-01 | 0.021 | 0.067 | 7.50E-01 | 0.109 | 0.072 | 1.28E-01 |
| rs12418451/A | -0.002 | 0.071 | 9.78E-01 | 0.021 | 0.054 | 6.95E-01 | 0.132 | 0.06 | 2.82E-02 |
| rs11228565/A | 0.041 | 0.08 | 6.09E-01 | 0.057 | 0.06 | 3.49E-01 | 0.159 | 0.068 | 1.90E-02 |
| rs10896450/G | 0.04 | 0.066 | 5.44E-01 | -0.044 | 0.05 | 3.82E-01 | 0.154 | 0.056 | 5.66E-03 |
| rs4054823/C | -0.041 | 0.065 | 5.31E-01 | -0.025 | 0.048 | 6.05E-01 | -0.062 | 0.055 | 2.57E-01 |
| rs11649743/A | -0.031 | 0.09 | 7.33E-01 | 0.022 | 0.064 | 7.27E-01 | -0.07 | 0.074 | 3.42E-01 |
| rs4430796/G | -0.042 | 0.066 | 5.19E-01 | -0.046 | 0.049 | 3.47E-01 | -0.136 | 0.055 | 1.32E-02 |
| rs1859962/G | 0.028 | 0.065 | 6.72E-01 | 0.004 | 0.05 | 9.42E-01 | 0.062 | 0.055 | 2.66E-01 |
| rs8102476/T | 0.101 | 0.07 | 1.46E-01 | 0.032 | 0.051 | 5.29E-01 | 0.039 | 0.058 | 5.06E-01 |
| rs2735839/A | -0.044 | 0.101 | 6.62E-01 | -0.252 | 0.071 | 4.33E-04 | -0.305 | 0.082 | 2.18E-04 |
| rs9623117/C | 0.056 | 0.081 | 4.86E-01 | -0.062 | 0.06 | 3.04E-01 | -0.057 | 0.068 | 3.98E-01 |
| rs5759167/T | -0.032 | 0.066 | 6.24E-01 | 0.009 | 0.051 | 8.63E-01 | -0.056 | 0.056 | 3.20E-01 |
| rs5945572/A | -0.037 | 0.048 | 4.51E-01 | 0.044 | 0.037 | 2.36E-01 | 0.025 | 0.041 | 5.38E-01 |
